# Rethinking Indian monsoon rainfall prediction in the context of recent global warming

**DOI:** 10.1038/ncomms8154

**Published:** 2015-05-18

**Authors:** Bin Wang, Baoqiang Xiang, Juan Li, Peter J. Webster, Madhavan N. Rajeevan, Jian Liu, Kyung-Ja Ha

**Affiliations:** 1 International Pacific Research Center and Department of Meteorology, University of Hawaii at Manoa, Honolulu, Hawaii 96825, USA; 2 Earth System Modeling Center, Nanjing University of Information Science and Technology, Nanjing 210044, China; 3 NOAA/Geophysical Fluid Dynamics Laboratory, Princeton, New Jersey 08540, USA; 4 University Corporation for Atmospheric Research, Boulder, Colorado 80307, USA; 5 Earth and Atmospheric Sciences, Georgia Institute of Technology, Atlanta, Georgia 303332, USA; 6 Earth System Science Organization, Ministry of Earth Sciences, Pune 411 008, India; 7 Key Laboratories for Virtual Geographic Environment and Numerical Simulation of Large Scale Complex System, School of Geography Science, Nanjing Normal University, Nanjing 210023, China; 8 Jiangsu Center for Collaborative Innovation in Geographical Information Resource Development and Application, Nanjing 210023, China; 9 Division of Earth Environmental System, Pusan National University, Busan 609-735, S. Korea

## Abstract

Prediction of Indian summer monsoon rainfall (ISMR) is at the heart of tropical climate prediction. Despite enormous progress having been made in predicting ISMR since 1886, the operational forecasts during recent decades (1989–2012) have little skill. Here we show, with both dynamical and physical–empirical models, that this recent failure is largely due to the models' inability to capture new predictability sources emerging during recent global warming, that is, the development of the central-Pacific El Nino-Southern Oscillation (CP–ENSO), the rapid deepening of the Asian Low and the strengthening of North and South Pacific Highs during boreal spring. A physical–empirical model that captures these new predictors can produce an independent forecast skill of 0.51 for 1989–2012 and a 92-year retrospective forecast skill of 0.64 for 1921–2012. The recent low skills of the dynamical models are attributed to deficiencies in capturing the developing CP–ENSO and anomalous Asian Low. The results reveal a considerable gap between ISMR prediction skill and predictability.

## Abstract (web summary)

Indian summer monsoon rainfall impacts millions of people and accurate forecasts are highly desirable, yet official operational forecasts have failed to predict extreme events in 1994, 2002, 2004 and 2009. Here, the authors use dynamical and physical–empirical models to investigate the reasons for this failure.

## Graphical abstract

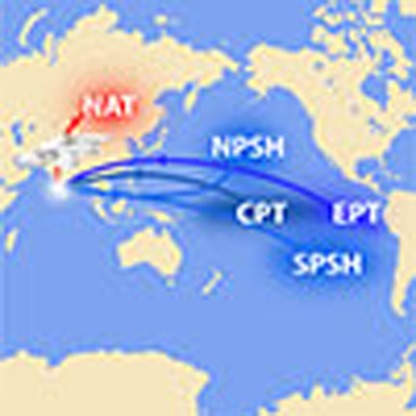

The Indian Meteorological Department (IMD) measures the Indian summer monsoon rainfall (ISMR) using the All-India Rainfall Index (AIRI), the total amount of summer June-to-September (JJAS) rainfall averaged over the entire Indian subcontinent^1^, which represents very well the leading principal mode of the ISMR^2^ and the like-signed severe rainfall anomalies occurring across India^3^. Swings in the AIRI, even with variations of only 10%, can cause severe flooding or drought, affecting Indian food production and security, and Gross Domestic Product^4^,^5^. Mitigation is possible with timely and accurate predictions of AIRI.

During the last century, great efforts have been made to understand the basic physics of the monsoon, with some success^6^,^7^,^8^,^9^,^10^,^11^. Prediction efforts started as early as 1886, but there has not been a similar or satisfactory success^1^,^7^,^12^,^13^,^14^. In fact, the correlation skill of the official operational forecasts made by IMD statistical models for 1989–2012 is −0.12 (Fig. 1). The negative skill is mainly due to the failure in forecasting four extreme events in 1994, 2002, 2004 and 2009. Why is the forecasting skill of the ISMR so poor in the recent decades? Does the Indian monsoon possess intrinsically limited predictability during the recent global warming period? Or is it because of the deficiencies in the methodologies used or in the models? Here we address these questions by examining 46-year multi-model ensemble (MME) dynamical hindcast and a 92-year retrospective forecast made by a physically motivated empirical prediction model. We show that the recent failure is largely due to the models' inability to capture new predictability sources emerging during the recent global warming.

## Results

### Performance of dynamical predictions

**Figure 1:**
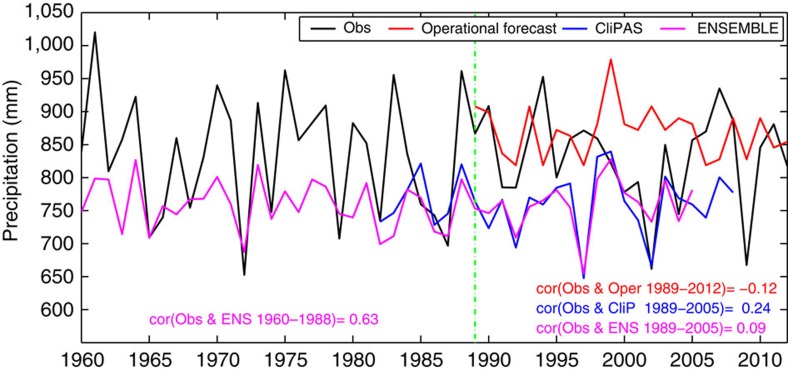
Time series of observed and predicted AIRI. Shown are the observed AIRI (units: mm) during 1960–2012 (Obs), predicted AIRI by IMD operational forecast (Oper) during 1989–2012, by four CliPAS dynamical models' ensemble during 1982–2008 (CliP) and by five ENSEMBLE dynamical models' ensemble prediction during 1960–2005 (ENS). The temporal correlation skills are shown at the bottom. The green vertical line indicates the year of 1989. The corresponding MSSS skills (see Methods) for operational, ENSEMBLE and CliPAS are, −0.36 (1989–2012), −1.32(1989–2005) and −1.36 (1989–2005), respectively.

The recent low skill of AIRI prediction is not only seen in operational statistical models but also found in dynamical predictions of the state-of-the-art global-coupled climate models. We have examined five climate models that participated in ENSEMBLE project^15^ for the 46 years (1960–2005) and four climate models that participated in the APCC/CliPAS project^16^ for the 27 years (1982–2008; see Methods). The hindcast correlation skill for the five ENSEMBLE models' MME is 0.09 for 1989–2005 and the four APCC/CliPAS models' MME skill is 0.24 for 1989–2005 (Fig. 1). Interestingly, the APCC/CliPAS MME and the ENSEMBLE models' MME have the similar performance: the correlation coefficient between the two MMEs' hindcasts of AIRI for 1989–2005 is 0.87, suggesting that the current dynamical models capture similar sources of predictability. Both MMEs also show systematic biases in the climatological mean and year-to-year variance compared with observations (Fig. 1), indicating the necessity for a mean bias correction and variance inflation in practical dynamic forecast.

However, we also find that the ENSEMBLE MME hindcast of AIRI has a good skill of 0.63 for the earlier period from 1960 to 1988 (Fig. 1). The results here imply that the recent failure of the ISMR prediction reflects secular changes in the prediction skills, a more reliable validation of the dynamical models' performance may need hindcast data longer than 40–50 years, and a more reliable estimation of the monsoon rainfall predictability may need centennial hindcast data.

### Physically motivated empirical model

**Figure 2:**
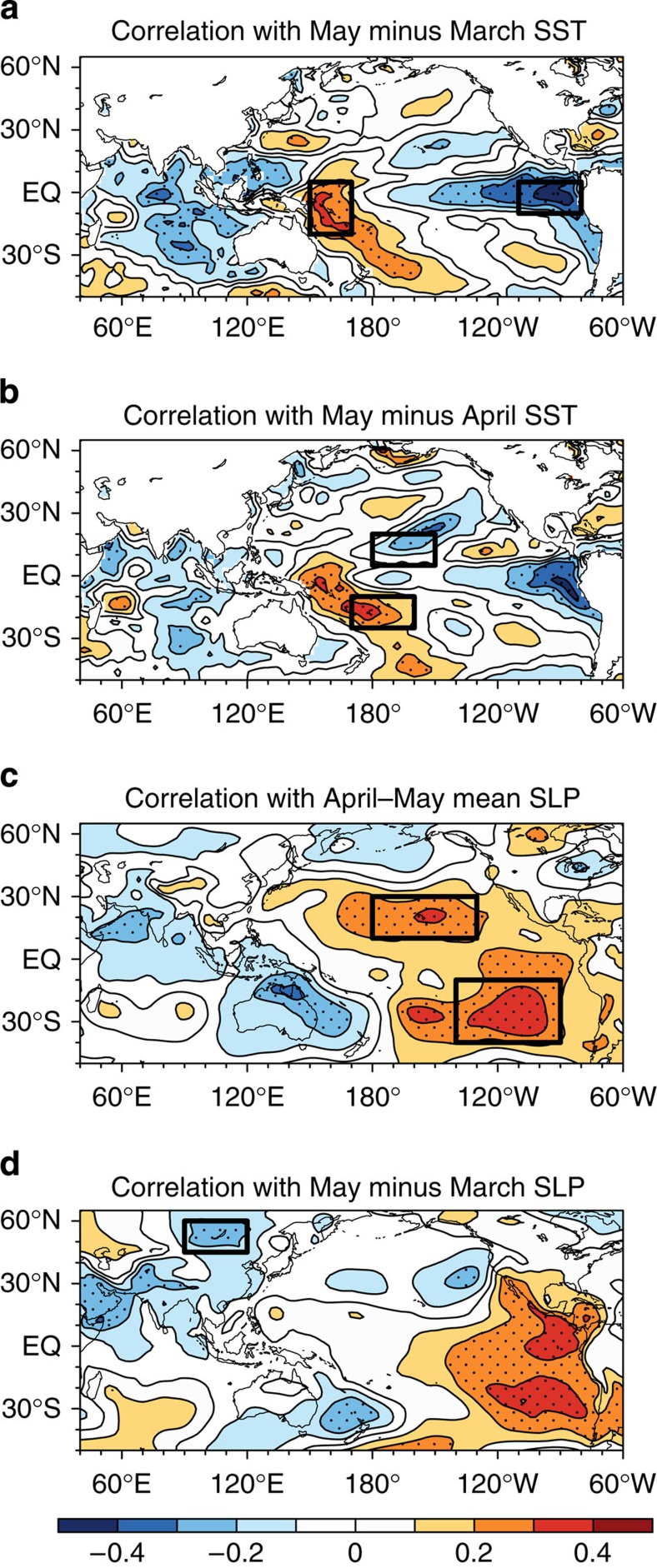
AIRI–predictor correlations in the observation for the period 1900–1988. The correlation coefficients between observed JJAS AIRI and (**a**) May minus March SST, (**b**) May minus April SST, (**c**) April–May mean SLP and (**d**) May minus March SLP. The black boxes outline the regions for the predictors defined in Table 1. Black dots in each panel represents the region with correlation significant at the 95% confidence level (Student's *t*-test).

To estimate the predictability of the AIRI using as-long-as-possible retrospective prediction, we developed a physically motivated empirical (P–E) model using the data from 1900 to 1988, and performed a 92-year independent retrospective prediction using data from 1871 to 2012 (see Methods). The P–E model was established based on an understanding of the physical processes linking predictors and the predictand. Statistical tests are used as an auxiliary tool to maximize the predictors–predictand correlation in training periods and to confirm their significance and ascertain mutual independence among the predictors. On the basis of physical considerations, rather than fishing statistical predictors, we confine the predictor search in only two fields, sea surface temperature (SST) and sea level pressure (SLP) and only two types of precursors, that is, persistence signals in spring and tendency signals across spring. The former often hint maintenance of anomalies due to positive atmosphere–ocean–land interaction processes, while the latter indicates the direction of the anomaly development. This physically motivated searching principle makes the selection procedure objective and easy to apply to other climate prediction problems. For AIRI, only four correlation maps were examined (Fig. 2); the April–May mean SST is not used owing to its high correlation with the April–May mean SLP.

### Mining new predictors for AIRI

**Figure 3:**
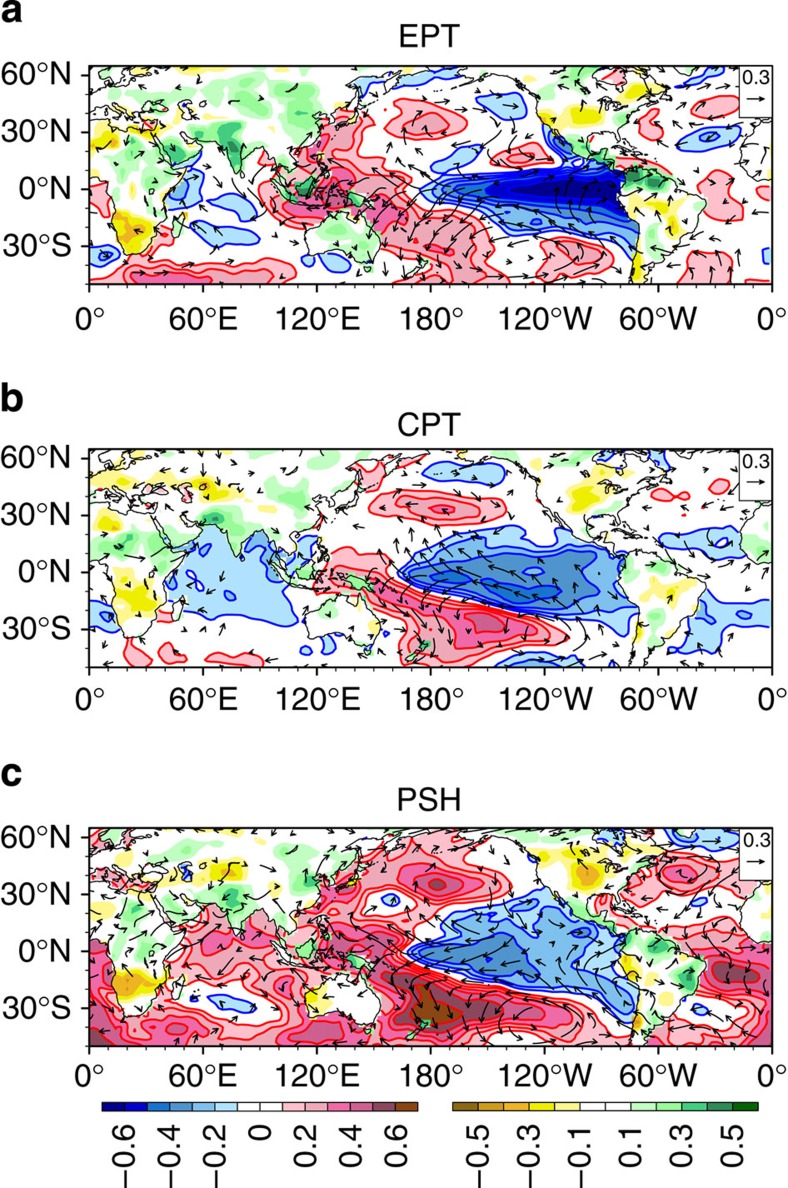
Three ENSO-related predictors. Shown are the correlation maps of the boreal summer (JJAS) SST anomalies (shading and contour over ocean) with respect to the predictor (**a**) EPT (EP–ENSO), (**b**) CPT (CP–ENSO) and (**c**) PSH (foreshadowing mega-ENSO) during 1900–1988. Also shown are those of land precipitation (shading over land) and 850 hPa winds (vectors) with respect to the predictors.

It is well known that the ISMR variations are primarily driven by eastern Pacific (EP) El Nino-Southern Oscillation (ENSO)^17^ through tropical teleconnection^6^,^9^,^10^,^11^. Traditionally, the April–May Nino 3 or Nino 3.4 SST anomaly was used as a persistent predictor. However, we use an east–west-oriented SST tendency as a new predictor because the EP–ENSO develops through Bjerknes feedback^18^, that is, the equatorial zonal wind-SST gradient feedback through changing oceanic thermocline depth and upwelling^19^. This positive Bjerknes feedback can be captured well by the Pacific east–west dipolar SST tendency from March to May (denoted by EPT, Table 1 and Fig. 2a). For instance, a cooling tendency during spring in the equatorial EP (and/or a warming in the equatorial western Pacific) signifies an enhanced east–west thermal contrast and associated pressure gradients in the ensuing summer, which enhances equatorial easterly that further strengthens upwelling in the EP, leading to development of an EP La Nina. Thus, EPT connotes a growing cold phase of EP–ENSO^18^ event in the ensuing summer (Fig. 3a). Note that the selected boxes also maximize the correlation of EPT with the AIRI. Other significant signals in Fig. 2a are not independent from the EPT.

Given the recent weakening of the AIRI and EP–ENSO relationship^20^, it remains a great challenge to find new sources of predictability that can foresee the pathway leading to development of the diversity of ENSO and the Asian land surface pressure anomalies.

The central-Pacific (CP)-ENSO^21^,^22^ has recently been found to have conspicuous impact on ISMR^23^ but no predictor has been proposed. A CP–ENSO develops mainly through zonal advection by ocean currents^22^. To capture this mechanism, we look for the north–south dipolar SST tendency in the CP (denoted by CPT, Table 1 and Fig. 2b) that signifies ensuing southern warming–northern cooling in the CP. The north–south SST contrast further strengthens northward pressure gradients and associated equatorial easterlies and westward ocean currents, leading to a cold phase of CP–ENSO (Fig. 3b). Considering the numbers of SST observations (Supplementary Fig. 1), the location of the northern box for CPT was slightly shifted eastward towards the CP ship tracks. Note that the selected northern box does not maximize the correlation, but it is selected because of its clear physical meaning. The May minus April SST tendency used here is better than May minus March tendency because the CP–ENSO tends to develop later in the spring and has a shorter life span compared with the EP–ENSO. Although the maximum correlation regions on this map are located in the equatorial western and EP, they were not selected because of their dependence on the EPT predictor.

The mega-ENSO^24^, which has a pattern similar to Interdecadal Pacific Oscillation^25^ but involves both interannual and multi-decadal variations of the Pacific basin-wide SST variability, has been recently identified as a major driver for the northern hemisphere monsoon rainfall variations including the ISMR^24^. The mega-ENSO involves an off-equatorial atmosphere–ocean thermodynamic feedback between the two Pacific subtropical highs (PSHs)/trades and basin-wide SST anomalies^26^. Thus, we define a PSH predictor (Table 1 and Fig. 2c) that gauges the anomalous intensity of the two PSHs during April and May that can foreshadow intensification of a mega-ENSO event (Fig. 3c). Thus, the predictor PSH serves as a mega-ENSO predictor. Coincidently, the two boxes in Fig. 2c also maximize the PSH correlation with AIRI. Note also that all three ENSO-related spring predictors are well correlated with not only the JJAS growing ENSO (Fig. 3) but also the corresponding mature ENSO anomalies in ensuing October through December (Supplementary Fig. 2).

In addition to the abovementioned three ENSO-related predictors, an enhanced ISMR is accompanied by an abnormal low pressure over Asian continent. To presage the development of abnormal Asian low, we focus on spring SLP over North Asia (NA). We found that the decreasing SLP tendency from March to May in the vicinity of Siberian High pressure centre near the Lake of Baikal (NAT, Table 1, Fig. 2d) represents a spring warming and low SLP anomaly over the entire Asian continent (Supplementary Fig. 3a). The largest correlation over the eastern tropical Pacific in Fig. 2d was not selected because of its high correlation with the predictor EPT. The NAT foreshadows the establishment of an anomalous Asian Low in the following summer that enhances the southwest monsoon rainfall over India (Supplementary Fig. 3b).

None of the above four predictors used in the present P–E model was not used by IMD prediction system (Supplementary Fig. 4). The three new ENSO-related predictors have distinctive periodicities (Supplementary Fig. 5): the EPT has quasi-biennial and quasi-quadrennial periodicities; the CPT has 3–4-, 8- and 20-year cycles; and the PSH has significant quasi-biennial and multi-decadal (60 year) peaks. Thus, they reflect different flavours of ENSO development that make complementary contributions to ISMR prediction on different timescales. The new land predictor NAT peaks on quasi-biennial and 10-year periodicities, which, to some extent, differs from the three ENSO-related predictors.

### Prediction skill of the P–E model

**Figure 4:**
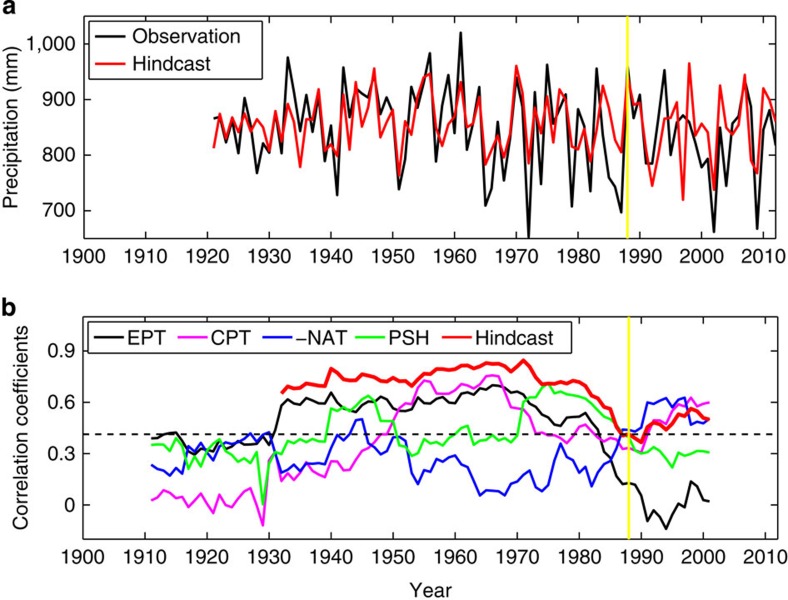
92-year independent hindcast and secular changes in the hindcast skill and AIRI–predictor relationships. (**a**) The observed (black) and the 92-year independent hindcast (red) AIRI with the P–E prediction model using the four predictors listed in Table 1. The correlation and MSSS skill (see Methods) are 0.64 and 0.40, respectively, for the period 1921–2012. (**b**) Time series of the 23-year central sliding independent hindcast skill (thick red) and the 23-year sliding correlation coefficients between the JJAS AIRI and each of the four predictors: EPT, CPT, reversed NAT and PSH. The statistical significant correlation coefficient at 95% confidence level (Student's *t*-test) is ±0.413 for the sample size of 23 which is indicated by the dashed lines. The symbols for the predictors are explained in Table 1. The yellow vertical line indicates the year of 1989.

The above four complementary predictors are physically connected to and significantly correlated with the AIRI using the data from 1900 to 1988 (Table 2). These predictors reflecting different physical processes are used to build prediction equations. Since reliable estimation of the practical predictability requires centennial independent retrospective forecasts, we developed a suite of progression (forward rolling) multi-regression prediction equations using the same four predictors (Table 1) and 142-year observations from 1871 to 2012. At each progression step, the prediction equation is derived using only 50-year training data and the AIRI is predicted for the ensuing 10 years (see Methods). These 10-year rolling predictions are ‘independent' in the sense that no ‘future' (after initial date of prediction) information is used beyond the training period. Starting from 1871, totally, 10 segments of 10-year predictions were made for the 92 years (1921–2012). The 92-year retrospective forecast correlation skill reaches 0.64 (Fig. 4a), and the truly independent forecast skill for 1989–2012 is 0.51, which is significantly higher than the IMD operational forecast skill (−0.12). The deterministic skill measure here also reflects the probabilistic measure as they are related^16^. Previous studies^27^,^28^ have reported maximum correlation skill of ∼0.5 from statistical and dynamical forecast models for shorter periods; thus, the skill here demonstrates a substantial improvement over past predictions.

As shown by Delsole and Shukla^28^, if all the data (model development and validation) are used to select the predictors, the cross-validated skill may be inflated. For the period of 1921–1950, the forecast used partial training information so that the skill of 0.68 in this period is possibly partially inflated. For the period of 1951–1988, the forecast used full training information so that the prediction skill of 0.74 during this period is likely more inflated. However, the independent forecast skill of 0.51 for the 1989–2012 is independent of training period data, thus not inflated.

### Causes of the low forecast skills in the recent decades

The recent 24 years are indeed a challenging period for ISMR prediction: the P–E model has a high skill of 0.77 for 1960–1988 but drops to 0.51 for 1989–2012 (Fig. 4a). This is mainly attributed to the drastic weakening of the AIRI–EPT predictor relationship (Fig. 4b). In Fig. 4b, a test of significance of running correlations^29^ was carried out. The running correlations between the predictor EPT (CPT) and AIRI are significantly more variable than expected from noise at 95% (90%) confidence level. The running correlations with PSH, NAT and the hindcast are insignificantly distinguishable from those expected from noise.

The AIRI–CPT relationship became significant after 1940 when the ocean observation improves (Supplementary Fig. 1). In the recent three decades, the AIRI–CPT relationship remains significant especially after the late 1990s when CP–ENSO became the dominant mode of ENSO^30^. It is notable that the land warming predictor (NAT) is complementary to EPT; its increasing skill in the recent decades tends to compensate the decreasing skill of EPT (Fig. 4b). Although the prediction skill is relatively low in the recent decades, the three new predictors (PSH, CPT and NAT) positively contribute to the improved skill.

**Figure 5:**
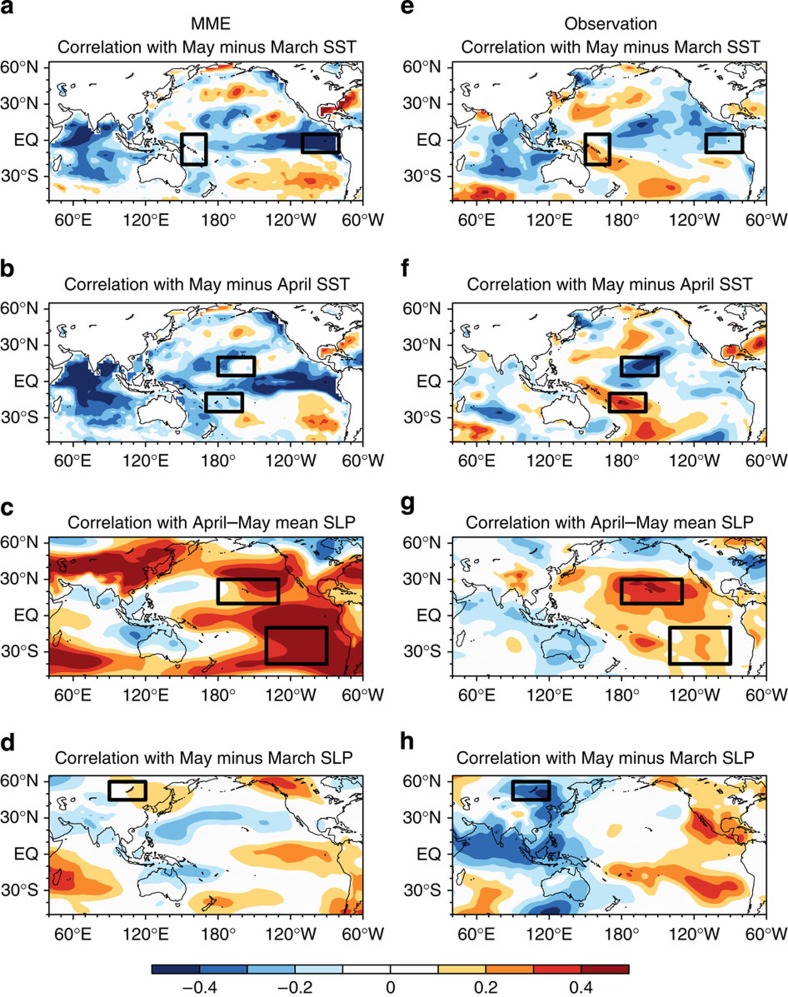
AIRI–predictor relationships in the dynamical MME and observation for the period 1960–2005. (**a**–**d**) The correlation maps between the JJAS AIRI in the five ENSEMBLE models' MME and (**a**) May minus March SST, (**b**) May minus April SST, (**c**) April–May mean SLP anomalies and (**d**) May minus March SLP. (**e**–**h**) are the same as in **a**–**d**, respectively, but for the observation. The black boxes outline the regions for the predictors defined in Table 1.

We further analysed the 46-year hindcast data made by the ENSEMBLE models to find why the dynamical MME hindcast skill sharply declined after the late 1970s. The results in Fig. 5 indicate that the ENSEMBLE models' MME captures the AIRI–EPT and AIRI–PSH relationship reasonably well but overestimate the former compared with the observed weakening relationship. However, the models totally missed the CP–ENSO and Asian Low (NAT) predictors. When the ENSO behaviour (amplitude, frequency, structure and evolution) changed in the late 1970s (refs 23, 31, 32), the dynamical models could not capture the declining role of the EP–ENSO and the increasing role of the CP–ENSO so that the modelled ENSO teleconnection patterns did not match observations^13^,^14^,^27^. In addition, the Asian Low predictor has significant contribution to the AIRI in the recent period (Fig. 4b), but the models have no capacity in catching up this tendency (Fig. 5d,h).

## Discussion

The 92-year hindcast skill (0.64) may provide an estimate for the lower bound of ISMR predictability, while at least 41% of the ISMR variance are predictable (see Methods), pointing to a significant room for improving current seasonal forecasts. While the dynamical models are steadily improving, the skill of the best model is still significantly below potential limit of predictability. There is a need to develop P–E models to possibly attain higher prediction skills and to help understanding the causes of the models' deficiencies.

Compared with previous statistical models, the superior prediction skill of the new P–E model arises primarily from use of the three new predictors, that is, predictors signifying developing CP–ENSO, enhancing PSHs and the abnormal Asian Low during spring. In addition, use of tendency predictors appears to provide more skillful predictions than those obtained with monthly anomalies^27^. Our 24-year (1989–2012) independent prediction provides a more rigorous validation than the ‘cross-validation' or any other existing validation method. We have also verified the model prediction for 2013 and 2014, which show the predictions being qualitatively correct. However, statistical validation of the real model prediction skills may require a decade long real-time prediction.

Prediction of severe drought and flood years of ISMR is important but remains a very challenging issue. For the 92-year forecast, the P–E model generally predicts the severe drought and flood years with correct sign but the amplitude of some events is underestimated and two false alarms occurred (Supplementary Fig. 6).

The P–E model can be extended to a wide range of climate predictability and prediction problems. It is primarily based on the physical understanding of the lead–lag linkage between the predictors and predictand. Although simultaneous relationships between a predictand and lower boundary anomalies have been studied for decades, the proposed understanding of the physical basis for lead–lag relationships represents advancement in climate study and it is more valuable for invention of prediction tools. We note that the dynamical models' low skill for a short period of 1989–2005 is not significantly different from a skill of 0.45 due to random sampling variability. Since the dynamical models have been significantly improved in the recent decade and will provide an ultimate prediction tool, an updated assessment of the current dynamical models' hindcast skill is needed. Our explanations of the physical meanings of the predictors should be viewed as hypotheses. Comprehensive numerical experiments with coupled climate models should be conducted to test these hypotheses.

We also note that the decrease in the recent AIRI prediction skill concurs with the most prominent recent global warming with the amplitude of 0.4 °C since the late 1970s (ref. 24). Figure 4b shows that since late 1970s, the decline of the AIRI–EPT relationship is unprecedented over the last century, and both the CP–ENSO and the Asian Low predictors have increased their correlations with AIRI. More frequent occurrence of CP–ENSO has been speculated for both anthropogenic origin^33^ and multi-decadal origin^30^. The secular change of the predictor–predictand relationships seems to be affected by both global warming and a multi-decadal natural variation, but the precise mechanisms require further studies.

## Methods

### Data sets used in this study

(i) All-India rainfall data provided by IITM (Indian Institute of Tropical Meteorology) for the period from 1871 to 2011 ( http://www.tropmet.res.in/static_page.php?page_id=53) and IMD for 2012 ( http://www.imd.gov.in/section/nhac/dynamic/mon2012.jpg) were used to derive prediction equations. The operational predicted AIRI was based on IMD data. The two data have very similar year-to-year fluctuation but have different seasonal means: the IITM mean is 850 mm, while the IMD mean is 890 mm. (ii) National Climatic Data Center's Extended Reconstructed Sea Surface Temperature (v3b) at 2° spatial resolution for the period 1871–2012 (ref. 34). (iii) The twentieth century reanalysis data for the 850 hPa wind, SLP and 2 m air temperature for the period 1871–2010 (ref. 35). (iv) The twentieth century-merged statistical analyses of historical monthly precipitation anomalies reconstructed data at 2.5° spatial resolution for the period from 1900 to 2008 (ref. 36). (v) To extend the 20C data, we also use European Centre for Medium-Range Weather Forecasts Re-Analysis (ERA-interim) data for SLP for period 2011–2012 (ref. 37). (vi) IMD's operational forecast results during 1989–2012 are obtained from http://www.imdpune.gov.in/research/ncc/longrange/Previouslongrange/pre1989-2012.html.

To ensure the reliability of oceanic observations, the SST predictors were basically chosen in the regions where island instrumental records are available or with relatively large number of monthly accumulative ship-by opportunity observations^31^ (Supplementary Fig. 1).

### Hindcast experiments of the dynamical models

One set of the dynamical models' hindcasts was derived from the ENSEMBLES project^15^. This data set consists of five state-of-the-art coupled atmosphere ocean circulation models and the hindcast was made for the period of 1960–2005. The five models are from the Euro-Mediterranean Center for Climate Change (CMCC-INGV) in Bologna, European Centre for Medium-Range Weather Forecasts (ECMWF), the Leibniz Institute of Marine Sciences at Kiel University (IFM-GEOMAR), Météo France (MF) and UK Met Office (UKMO). AIRI in dynamical models is defined as the total land rainfall over India, which is different from the previous study that calculates the total rainfall in a box domain (70 E–90 E, 10 N–25 N)^27^. Another four state-of-the-art-coupled models' hindcast results (1982–2008) used in this paper, are adopted from the Climate Prediction and its Application to Society (CliPAS) project^16^ sponsored by the Asia-Pacific Economic Cooperation (APEC) climate center (APCC). The models include NCEP CFS version 2, ABOM POAMA version 2.4, GFDL CM version 2.1, and FRCGC SINTEX-Fmodel.

### The 92-year retrospective forecast

The progressional prediction models were built by using only the 50-year data as training period each step to build a four-predictor multiple-regression model. For example, we built the first prediction model using the training data of 1871–1920 to forecast ISMR for the next 10 years (1921–1930), and then built the second prediction model using the 1881–1930 data to forecast the next 10 years (1931–1940). The predictions for the rest years were done in the same manner. The retrospective forecast does not use any ‘future' data beyond the training period. We have also used 30 or 40 years as a training period at each step to reconstruct the empirical prediction models and to use 1, 5 and 10 years as the forecast periods. It is found that using 50 years as a training period is systematically better than 40 years and 30 years in dealing with the secular variations of the predictor–predictand relationships; and use of different forecast period ranging from 1 to 10 years has no significant impact on the forecast skill (Supplementary Table 1). We also used longer training period of 60 years, the forecast skill is almost the same as 50 years.

### The time of the forecast

Using our P–E model, a forecast of the ensuing AIRI can be made as early as 26 May, because the May predictors can be estimated with some surety 5 days before the end of the month by using short-range forecasts. The dynamic model forecast we used here is starting from 1 May, and the operational forecast starts from 1 June.

### Mean square skill score

In addition to the correlation skill, we also used the Mean Square Skill Score^38^ (MSSS) to measure the deterministic forecasts skill in Fig. 1. For the 92-year retrospective forecast, the MSSS score is 0.41. The MSSS is defined as follows:


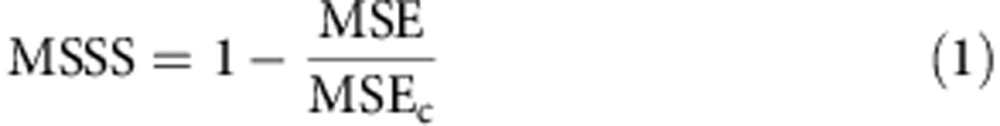


Where the mean squared error of the forecasts is:


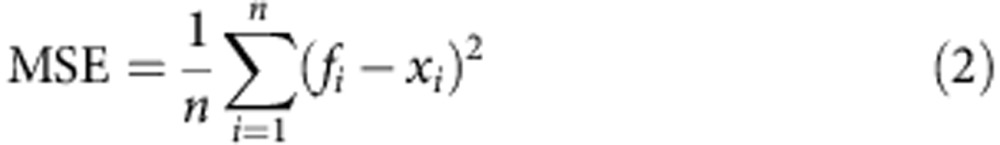


where *x* and *f* denote time series of observations and forecasts. The MSE for climatology is given by:


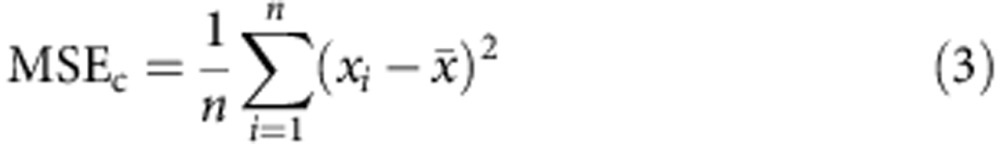


## Additional information

**How to cite this article:** Wang, B. *et al.* Rethinking Indian monsoon rainfall prediction in the context of recent global warming. *Nat. Commun.* 6:7154 doi: 10.1038/ncomms8154 (2015).

## Supplementary Material

Supplementary Information Supplementary Figures 1-6 and Supplementary Table 1

## Figures and Tables

**Table 1:** Definitions of the four AIRI predictors.

**Name**	**Definition**	**Meaning**
EPT	May minus March east–west SST dipole tendency: DSST* (20° S–5° N, 150° E-170° E) minus DSST (10° S–10° N, 110° W–80° W).	EP–ENSO predictor
CPT	May minus April SST north–south dipole tendency: DSST†(10°–25° S, 170° E–160° W) minus DSST (5°–20° N, 180°–150° W)	CP–ENSO predictor
PSH	April–May mean SLP averaged over SP (40° S–10° S, 160° W–90° W) and NP (10° N–30° N, 180°–130° W)	mega-ENSO predictor
NAT	May minus March SLP averaged over (45° N–60° N, 95° E–125° E)	Anomalous Asian Low predictor

AIRI, All-India Rainfall Index; CPT, SST tendency in central-Pacific; ENSO, El Nino-Southern Oscillation; EP, eastern Pacific; EPT, SST tendency in eastern-Pacific; PSH, Pacific subtropical high; SLP, sea level pressure

^*^Here DSST means the difference of SST between May and March (May minus March).

^†^Here DSST means the difference of SST between May and April (May minus April).

**Table 2:** Cross-correlation coefficients between AIRI and predictors during 1900–1988.

	**AIRI**	**EPT**	**CPT**	**PSH**	**NAT**
EPT	**0.51**		*0.22*	*0.22*	0.10
CPT	**0.35**	*0.22*		0.16	−0.04
PSH	**0.40**	*0.22*	0.16		−0.20
NAT	*−0.24*	0.10	−0.04	−0.20	

The statistical significance and mutual independence of the four predictors are summarized. The bold (Italic) numbers denote statistically significant at 99% (95%) confidence level by Student's *t*-test.
