# Supplementary material for: Rethinking Indian monsoon rainfall prediction in the context of recent global warming
Source: Nat Commun. 2015 May 18;6:7154. doi: 10.1038/ncomms8154 (PMC4479044; doi:10.1038/ncomms8154)
Supplement: Supplementary Information — Supplementary Figures 1-6 and Supplementary Table 1 [file ncomms8154-s1.pdf]

Supplementary information

Supplementary Figures:

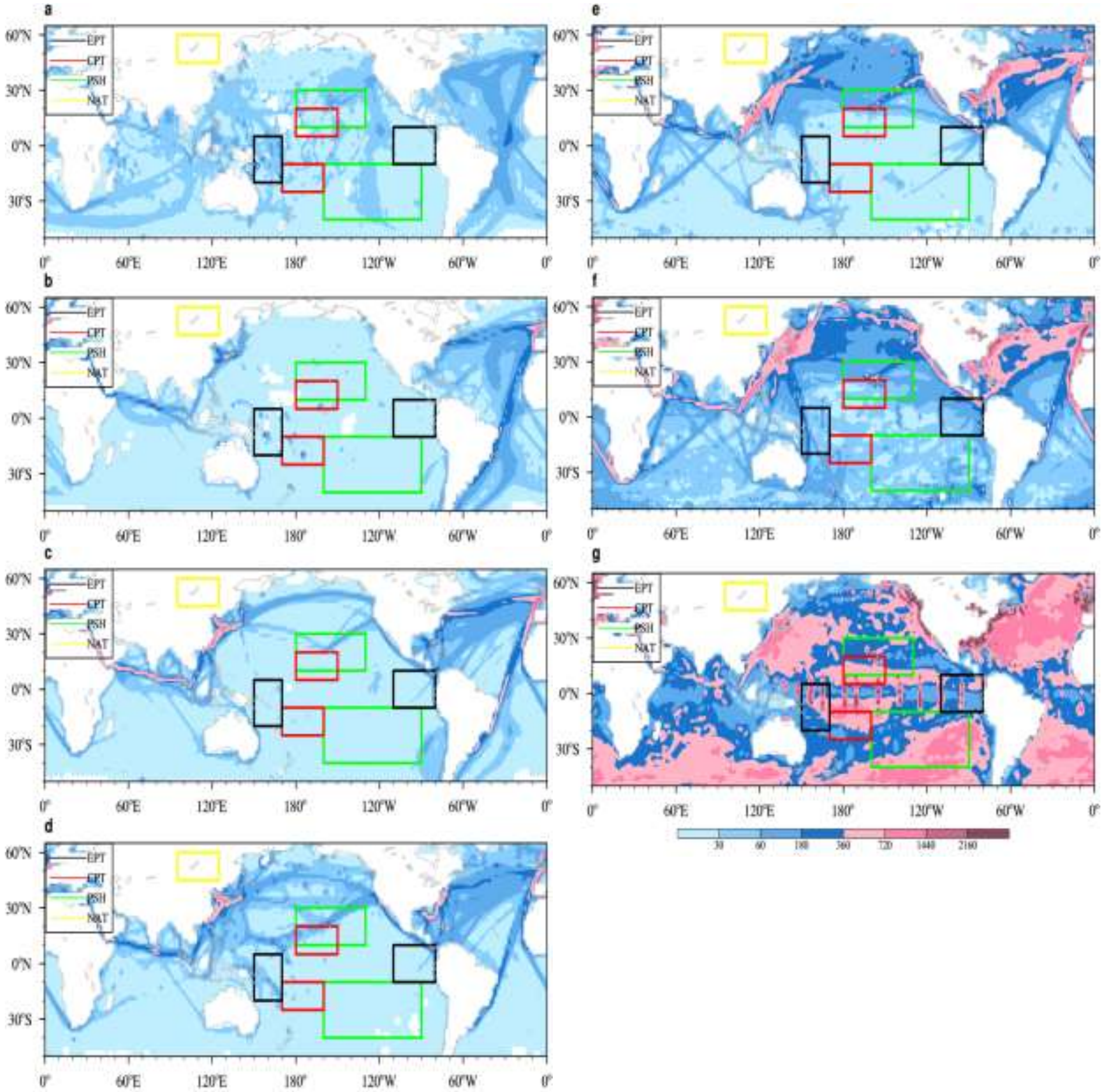

Supplementary Figure 1| Schematic diagram of the monthly accumulated numbers of SST observations. Accumulated numbers of SST observations during (**a**)1871-1890, (**b**)1891-1910, (**c**)1911-1930, (**d**)1931-1950, (**e**) 1951-1970, (**f**) 1971-1990, (**g**) 1991-2010. The boxes denote the locations of four predictors. The data are interpolated into 5*5 degree grid box.

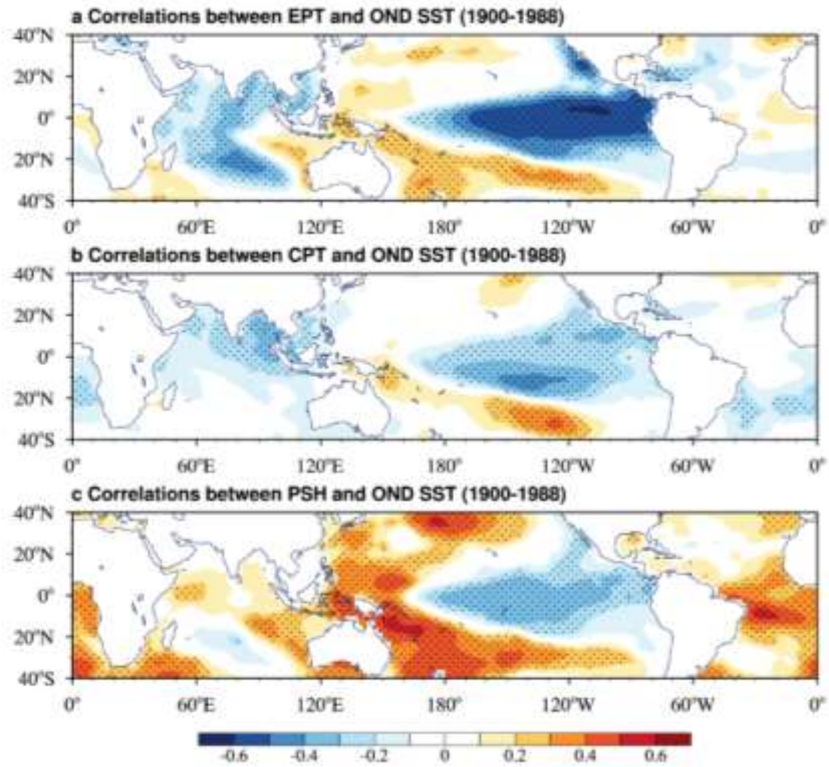

Supplementary Figure 2| Correlation map between three ENSO-related predictors and OND SST anomalies. (**a**) Correlations between EPT and OND SST anomalies during 1900-1988. (**b)** and (**c)** are same as in (**a**), but for CPT and PSH, respectively. Dots indicate regions where the correlation coefficients are significant at 95% confidence level (Student's t-test).

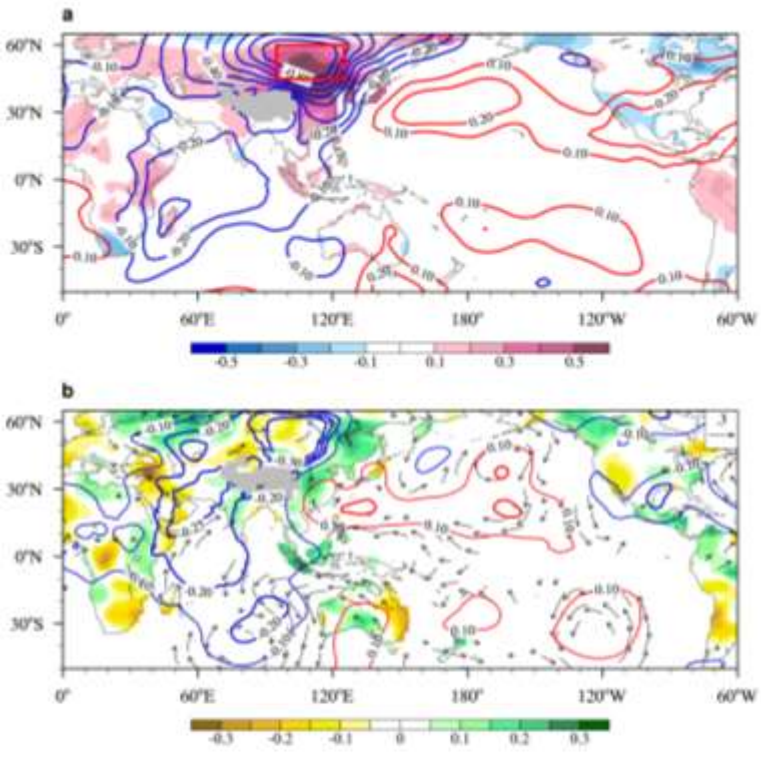

Supplementary Figure 3| Anomalies associated with the predictor NAT. (**a**) The correlation between reversed NAT and March-April-May mean 2 m air temperature over land (shading) and sea level pressure (contour) during the period of 1900-1988. The red box denotes the location where the predictor NAT is defined. (**b**) The correlation between reversed NAT and JJAS SLP (contours), land precipitation (shading) and 850 hPa winds (vector) during 1900-1988.

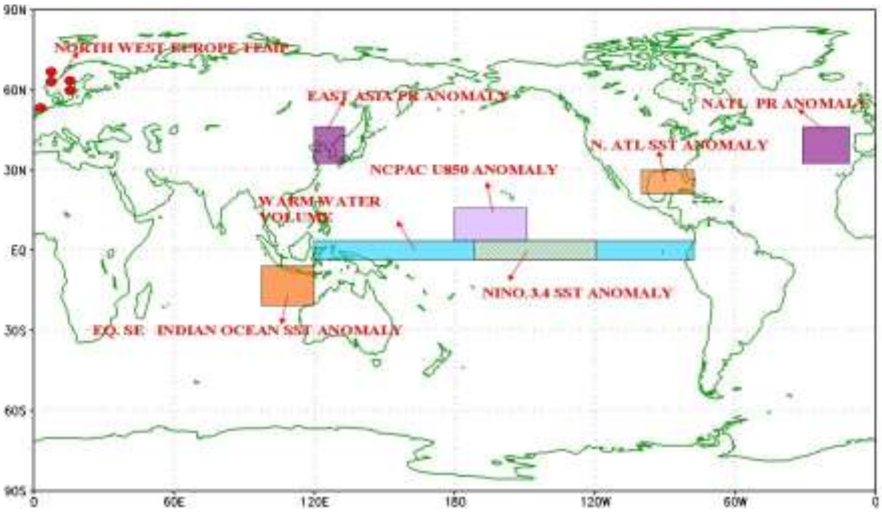

Supplementary Figure 4| List of predictors used in the IMD's operational forecast model[1].

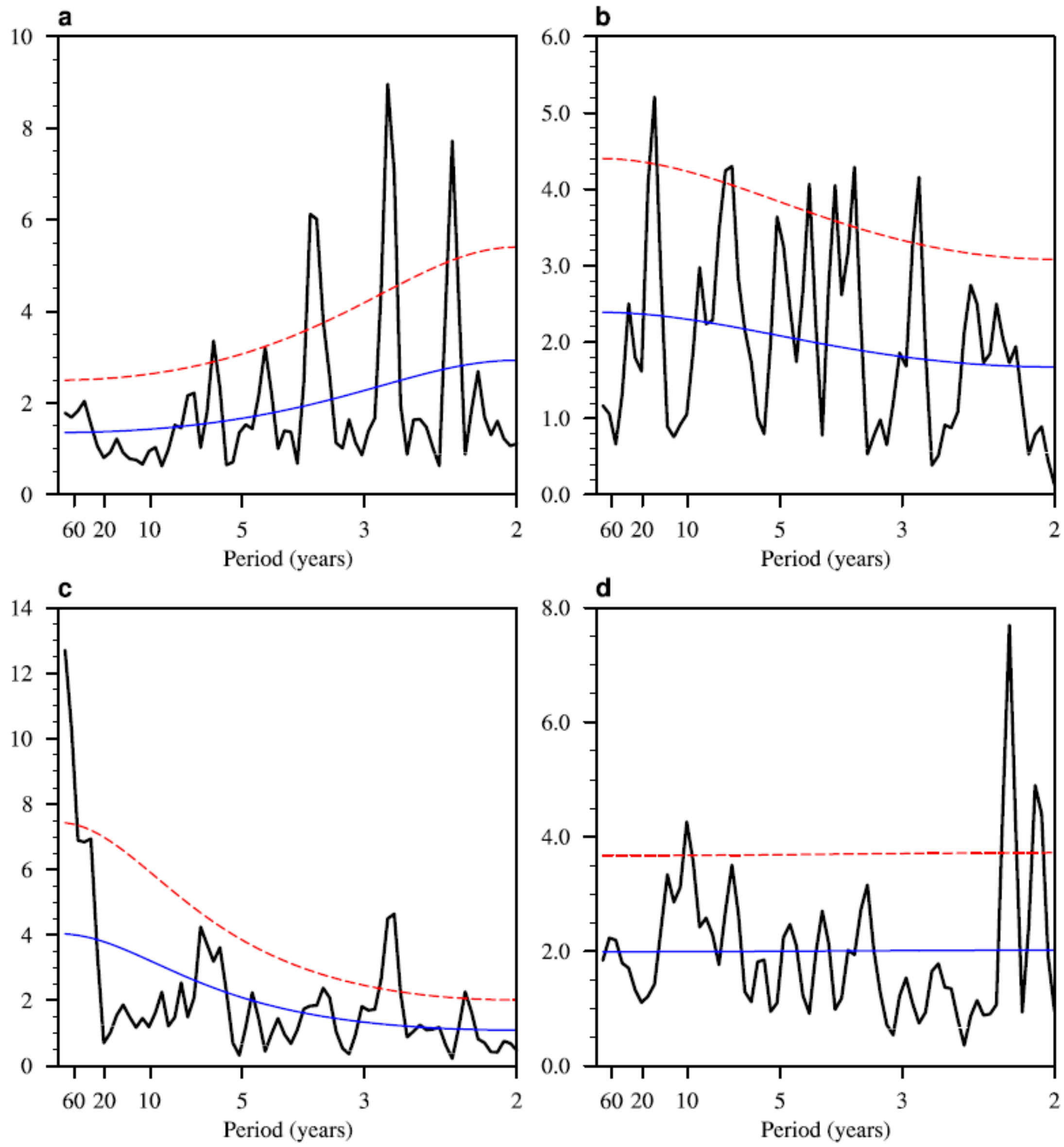

Supplementary Figure 5| Power spectra of four predictors. The spectra of (**a**) EPT, (**b)** CPT, (**c**) PSH and (**d**) NAT. The black line indicates spectrum, the blue line indicates "red noise" and the red line indicates the 90% confidence bounds.

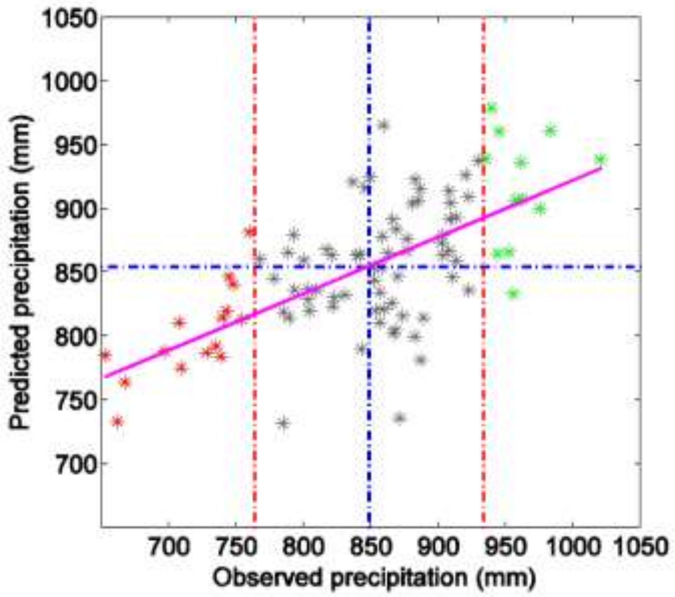

Supplementary Figure 6| The scatter diagram of observed AIRI and retrospective prediction of AIRI. The severe drought and flood years are shown by red and green, which are defined by the observed deviations exceeding ±10% (the two vertical red lines) of the long-term average of 848 mm (the vertical blue line). The correlation skill is 0.64 for the period 1921-2012.

Supplementary Table

Supplementary Table 1| Sensitivity of the retrospective prediction skills to the training period and hindcast period.

50yr-1yr	50yr-5yr	50yr-10yr
0.64	0.63	0.64

40yr-1yr	40yr-5yr	40yr-10yr
0.62	0.61	0.61

30yr-1yr	30yr-5yr	30yr-10yr
0.60	0.60	0.59

The forecast skill is measured by the correlation coefficient between observation and progression forecast during 1921-2012. Symbol “50yr-1yr” means training period is 50 years and the forecast period is 1 year.
